# Key immune checkpoint inhibitor-associated toxicities: from molecular mechanisms to clinical management

**DOI:** 10.3389/fphar.2026.1810121

**Published:** 2026-04-21

**Authors:** Tao Lu, Xiaoyan Song, Tianhong Zhang, Weiping Ge

**Affiliations:** Department of Gynecology, Qingdao Municipal Hospital, Qingdao, Shandong, China

**Keywords:** immune checkpoint inhibitors, immune dysregulation, immune-related adverse events, T-cell tolerance, toxicity management

## Abstract

Combination regimens built on immune checkpoint inhibitors (ICIs) can deepen antitumor immunity but also reveal a key translational bottleneck: how to escalate immune pressure while preventing immune-related adverse events (irAEs). IrAEs arising in combination therapy are framed as systems-level tolerance failures, driven by cytokine-network rewiring, myeloid–T cell feedback, antigen spreading, and tissue-resident immune programs that shape organ-specific toxicity phenotypes. Our distinctive contribution is an integrative framework that maps these mechanisms to measurable readouts and clinical decision points. We highlight pragmatic systems-immunology strategies to separate productive antitumor activation from early off-tumor inflammation and enable risk stratification across trials and real-world practice. This mini-review consolidates organ-focused recognition and severity-adapted management principles and discusses toxicity-mitigation approaches designed to preserve anticancer efficacy. We also outline reporting standards to harmonize toxicity phenotyping and endpoints across combination studies. Together, this synthesis connects combination design with deployable monitoring and prevention concepts for safer, more effective immunotherapy.

## 1 Introduction

Immune checkpoint inhibitors (ICIs), targeting immune regulatory molecules such as cytotoxic T lymphocyte-associated antigen 4 (CTLA-4), programmed cell death protein 1 (PD-1), its ligand PD-L1, and lymphocyte activation gene 3 (LAG-3), have substantially reshaped the therapeutic landscape of oncology (Ribas and Wolchok, 2018; Chen and Cheng, 2025). By releasing the natural brakes on T cells, ICIs enhance antitumor immune responses, leading to substantial survival benefits across multiple malignancies, including melanoma, lung cancer, and renal carcinoma (Reck et al., 2016). At the same time, the same mechanisms that enable potent tumor immunity can inadvertently disrupt immune self-tolerance, resulting in irAEs (Eldani et al., 2024). Moreover, ICIs have been increasingly applied in gynecologic malignancies, such as ovarian and endometrial cancers, as well as in breast cancer (Song et al., 2024; Knisely et al., 2024). Although clinical benefits are emerging, the risk of immune-related toxicities in these populations warrants careful evaluation, particularly in patients receiving combination regimens. Clinically, irAEs present as immune-mediated inflammation affecting multiple organ systems, often with organ-predominant phenotypes such as myocarditis, pneumonitis, and hepatitis. Their severity can range from mild to life-threatening, potentially limiting treatment continuity, dose intensity, and long-term benefit in a subset of patients (Schneider et al., 2021; Wolchok et al., 2017).

Recent research has shown that irAEs originate from a complex interplay of genetic predispositions, impaired central and peripheral immune tolerance, autoreactive T cell expansion, macrophage-mediated inflammation, and dysregulated cytokine networks (Wang et al., 2023; Esfahani et al., 2020; Chin et al., 2022; Barlas et al., 2024). Although advances have been made in clinical recognition and management, major gaps remain, including defining patient susceptibility, refining diagnostic criteria, and developing mechanism-guided therapeutic interventions (Wang et al., 2023; Naidoo et al., 2023; Keam et al., 2024). This review synthesizes the latest insights into the molecular basis, clinical manifestations, and emerging targeted management strategies for ICI-associated toxicities, with the goal of informing clinically actionable strategies for safer, more effective immunotherapy approaches.

## 2 Spectrum of ICI-associated toxicities

ICIs have revolutionized cancer treatment but frequently induce a wide spectrum of irAEs affecting multiple organ systems. Among these toxicities, cardiovascular complications, notably myocarditis, represent rare but serious conditions, characterized clinically by chest pain, dyspnea, and potentially fatal arrhythmias (Schneider et al., 2021; Mahmood et al., 2018). Although myocarditis occurs infrequently (<2%), it is associated with high mortality rates, particularly when ICIs are administered in combination (Inno et al., 2024). Pulmonary toxicities, including pneumonitis, manifest as interstitial lung disease and respiratory distress, and represent another significant ICI-related complication that may necessitate discontinuation of therapy (Rapoport et al., 2021; Guzel et al., 2025; Hong et al., 2023).

Gastrointestinal adverse events are among the most commonly reported ICI-related toxicities and include colitis and hepatitis (Gao et al., 2025). Symptoms range from mild abdominal discomfort to severe diarrhea, hepatocellular injury, and fulminant hepatic failure. Similarly, neurological and neuromuscular complications such as myositis and myasthenia gravis, although less common, significantly impact patient quality of life and overall survival (Plomp et al., 2025). Endocrine toxicities, like thyroiditis, adrenal insufficiency, and diabetes mellitus, frequently necessitate lifelong hormone replacement (Wright et al., 2021). Furthermore, cutaneous adverse events, such as rash and pruritus, are among the earliest and most prevalent irAEs, though typically milder in clinical severity (Sun et al., 2025). This wide spectrum of toxicities underscores the systemic nature of immune dysregulation triggered by checkpoint blockade. Consequently, recognizing the heterogeneous clinical manifestations, understanding underlying pathophysiological mechanisms, and establishing predictive biomarkers, such as autoantibody profiles, circulating cytokines, and peripheral immune cell signatures (Liang et al., 2024; Les et al., 2023), are critical for effective management of these toxicities (Keam et al., 2024). Notably, clinical trials in breast, ovarian, and uterine cancers have also reported overlapping patterns of irAEs, ranging from endocrine dysfunction to severe colitis (Cortes et al., 2022; Matulonis et al., 2019; Oaknin et al., 2022). These trials suggest that gynecologic cancer cohorts exhibit irAE patterns broadly consistent with those reported across tumor types. However, irAE risk and clinical course may still vary according to prior therapies, baseline comorbidities, and the use of combination regimens. For example, pre-existing autoimmune disease, prior thoracic irradiation, or concurrent ICI-based combinations may increase the likelihood or severity of organ-specific toxicities, supporting the need for risk-adapted monitoring and organ-focused, rather than cancer-type-specific, management strategies.

## 3 Molecular mechanisms underlying ICI-associated toxicities

**FIGURE 1:**
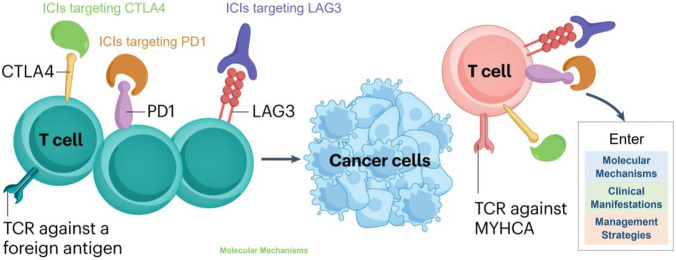
Schematic illustration of the mechanisms underlying ICI-associated toxicities. Central tolerance breakdown in the thymus, peripheral tolerance disruption via checkpoint blockade, autoreactive T-cell expansion, immune cell cross-talk, and cytokine/chemokine dysregulation collectively drive organ-specific inflammatory damage. This figure highlights the interplay of T cells, macrophages, and cytokine networks in mediating irAEs, providing a mechanistic framework for organ-predominant immune injury phenotypes and informing rational, targeted escalation strategies.

The molecular mechanisms underlying ICI-associated toxicities are complex and interrelated. Figure 1 provides an overview of these processes, illustrating how central and peripheral tolerance disruption, autoreactive T-cell expansion, immune cell cross-talk, and cytokine/chemokine dysregulation synergistically contribute to organ-specific damage. Central tolerance failure allows autoreactive T cells to escape thymic deletion, while peripheral checkpoint blockade unleashes excessive effector T-cell activity. These activated T cells interact with macrophages and other immune cells, amplifying inflammation through cytokine and chemokine cascades such as IFNγ–CXCL9/10 signaling (Munir et al., 2024; Ma et al., 2024). The figure highlights how these processes converge to drive tissue-specific immune damage, thereby providing a mechanistic framework that links immune dysregulation to organ-specific tissue injury and organ-predominant clinical phenotypes. To preserve clarity and readability, organ-specific biomarker links and decision-making details are presented in the organ-based sections and in Table 1 rather than being embedded in the schematic itself.

**TABLE 1:** Clinical spectrum, diagnostic features, guideline-consistent initial management, and selected steroid-refractory or investigational escalation options for immune checkpoint inhibitor-associated toxicities.

Organ system	Common irAEs	Typical culprit regimens (examples)	Key diagnostics	Initial management (standard initial care)	Steroid-refractory/targeted add-ons (examples)	Refs
Cardiov-ascular	Myocarditis, pericarditis	Dual ICI nivolumab + ipilimumab; PD-1/PD-L1 monotherapy (pembrolizumab, nivolumab, atezolizumab)	ECG, hs-troponin/CK, cardiac MRI; ± EMB	High-dose corticosteroids	Abatacept (CTLA-4-Ig); ruxolitinib (IFNγ–JAK/STAT program); ± IVIG/PLEX; guideline-directed HF/arrhythmia care	Johnson et al. (2016), Dougan et al. (2021)
Pulmon-ary	Pneumonitis	Pembrolizumab, nivolumab, atezolizumab (±chemo/anti-VEGF)	HRCT; bronchoscopy/BAL to exclude infection	Corticosteroids (grade-based)	Mycophenolate mofetil; selected cases tocilizumab (IL-6R)	Dougan et al. (2021), Haanen et al. (2022)
Gastroint-estinal	Colitis/enterocolitis	Ipilimumab; nivolumab + ipilimumab; PD-1 monotherapy	Stool pathogen panel; CRP/fecal calprotectin; colonoscopy + biopsies	Rehydration + corticosteroids	Infliximab (avoid in severe hepatitis/HF); vedolizumab (gut-selective, favored with hepatitis); ± JAK inhibitor where IFNγ-skewed	Abu-Sbeih et al. (2018)
Hepatobil-iary	Hepatitis	Ipilimumab, pembrolizumab/nivolumab; atezolizumab + bevacizumab (HCC)	Viral hepatitis work-up; autoantibodies; liver biopsy if uncertain	Corticosteroids	Mycophenolate mofetil → tacrolimus if needed; avoid infliximab	Peeraphatdit et al. (2020)
Endocrine	Thyroiditis, hypophysitis, adrenal insufficiency, diabetes	PD-1/PD-L1: thyroiditis (pembro/nivo/atezo); CTLA-4: hypophysitis (ipilimumab)	Axis labs (TSH/FT4; AM cortisol/ACTH); ± pituitary MRI	Hormone replacement (levothyroxine; stress-dose hydrocortisone, etc.)	Short steroid course only for florid gland inflammation; immunosuppression generally not required long-term	Barroso-Sousa et al. (2018)
Neuromu-scular	Myositis, MG (± myocarditis overlap)	More frequent with nivolumab + ipilimumab; also PD-(L)1	CK + troponin; EMG; AChR/MuSK Ab; muscle ± cardiac MRI/biopsy	High-dose corticosteroids	IVIG or plasmapheresis; rituximab in refractory humoral-leaning disease; coordinate cardio-neuro care	Guidon et al. (2021)
Dermatol-ogic	Maculopapular rash, pruritus; bullous disorders	Any ICI; CTLA-4 and combinations higher risk for severe forms	Dermatology exam; skin biopsy if severe/atypical	Topical steroids/antihistamines → systemic steroids if severe	For bullous/pemphigoid-like: rituximab or dupilumab in select cases per specialist	Tay et al. (2017)
Renal	Interstitial nephritis (AIN)	Predominantly PD-1/PD-L1	Creatinine/UA; consider renal biopsy	Corticosteroids; supportive care	Mycophenolate mofetil or azathioprine in persistent cases after biopsy confirmation	Alouani et al. (2023), Esfahani et al. (2019)

Initial management refers to guideline-consistent first-line care. Escalation options are provided as illustrative examples for steroid-refractory, organ-specific, or selected high-severity settings and should not be interpreted as uniformly established standard therapies across all irAEs.

### 3.1 Central tolerance breakdown

Central immune tolerance involves the elimination of autoreactive T cells during thymic selection, ensuring immune tolerance to self-antigens (Xing and Hogquist, 2012). Critical to this process is the expression of tissue-restricted antigens within thymic epithelial cells, mediated by the autoimmune regulator (AIRE) gene (Anderson et al., 2002). Insufficient or aberrant expression of certain self-antigens, such as cardiac myosin heavy chain alpha (MYHCA), allows autoreactive T cells to evade negative selection, thereby escaping thymic deletion (Lv et al., 2011). These T cells subsequently circulate and can target normal tissue antigens upon peripheral activation, particularly under conditions of immune checkpoint blockade, leading to autoimmune manifestations, including myocarditis and neuromuscular toxicities.

### 3.2 Peripheral tolerance disruption

Peripheral tolerance involves mechanisms that prevent activation and proliferation of autoreactive T cells outside the thymus. Immune checkpoints such as CTLA-4, PD-1, and LAG-3 are crucial regulators in maintaining this peripheral tolerance by suppressing T cell activation and promoting regulatory T cell (Treg) function (Munir et al., 2024). ICIs disrupt peripheral tolerance by blocking these checkpoint interactions, leading to unchecked activation and expansion of effector T cells (Murga-Zamalloa et al., 2020). This blockade significantly enhances antitumor immunity but simultaneously increases the risk of autoimmunity, enabling autoreactive T cells previously held in check to mediate tissue-specific inflammatory damage.

### 3.3 Immune cell activation and cross-talk

Emerging evidence highlights a critical role for immune cell crosstalk in driving ICI-associated toxicities. Blockade of PD-1-PD-L1 or CTLA-4 leads to pronounced activation and clonal expansion of autoreactive cytotoxic CD8^+^ T cells, which mediate direct tissue injury through release of cytotoxic effectors such as perforin and granzymes (Won et al., 2022). Macrophages, particularly CXCL9/CXCL10-expressing subsets, are activated via IFNγ produced by effector T cells, further amplifying local inflammatory responses (Ma et al., 2024). Beyond direct cytotoxicity, a “feed-forward” circuit often emerges in target tissues: activated T cells induce chemokines (e.g., CXCL9/CXCL10) that recruit additional effector cells, while myeloid cells reciprocally sustain T-cell activation through antigen presentation, costimulation, and inflammatory mediators. Tissue-resident memory-like T-cell programs and epitope spreading may further broaden the repertoire of self-reactivity, helping to explain why some irAEs persist or recur even after ICI discontinuation.

Humoral and innate compartments can also participate in these cross-talk loops. Increasing data support a role for B-cell activation and T–B interactions in selected irAEs, including expansion of autoreactive B-cell subsets and/or autoantibody-associated inflammation, which may complement T-cell–dominant injury patterns (Dhodapkar et al., 2023; Ghosh et al., 2022). In parallel, barrier-tissue toxicities (e.g., colitis, dermatitis) can involve type 3 immune features, with IL-17A–biased CD4^+^ responses and associated myeloid recruitment reported in clinical multi-omics analyses (Thomas et al., 2024), providing an additional axis of T-cell–myeloid cross-regulation in certain patients (Dimitriou et al., 2024). Additional immune cells, including natural killer (NK) cells, B cells, and dendritic cells, may also contribute to the immunopathology, although their exact roles in ICI-induced toxicity require further investigation (Wang et al., 2023).

### 3.4 Cytokine and chemokine networks

Cytokines and chemokines play pivotal roles in propagating ICI-mediated immune toxicities. IFNγ, a key cytokine produced by activated T cells, initiates and sustains inflammatory responses by signaling through the JAK-STAT pathway, promoting macrophage activation and inflammatory chemokine (CXCL9/CXCL10) secretion (House et al., 2020). These chemokines attract additional activated CXCR3-expressing T cells, resulting in a positive feedback loop of inflammation. Blocking these pathways, for example, with JAK inhibitors such as ruxolitinib, has shown potential in alleviating severe cases of toxicity in preclinical and clinical studies (Nguyen et al., 2022). Understanding these complex signaling networks provides valuable insights for developing targeted interventions aimed at mitigating toxicities without impairing antitumor immunity.

## 4 Clinical manifestations and diagnosis

The clinical presentation of ICI-associated toxicities is highly variable, reflecting the widespread distribution of immune checkpoint molecules and their functions across different organ systems. Recognizing these toxicities early is critical, as timely identification and intervention can significantly improve clinical outcomes and survival. Although some irAEs are rare but life-threatening, the most prevalent toxicities in day-to-day practice typically involve the gastrointestinal, endocrine, and dermatologic systems and therefore merit particular attention in clinical monitoring frameworks.

### 4.1 Cardiovascular system—myocarditis under dual ICI as a paradigm

Myocarditis occurs most prominently with dual checkpoint blockade (such as nivolumab + ipilimumab), though it can arise with PD-1/PD-L1 monotherapy or chemoimmunotherapy (Johnson et al., 2016). Patients typically present early with chest pain, dyspnea, conduction disease, or malignant arrhythmias, and may exhibit an overlap with myositis/myasthenia gravis. Screening during early treatment cycles should include frequent electrocardiograms (ECG) and serial high-sensitivity troponin (hs-troponin) with creatine kinase (CK). Cardiac magnetic resonance imaging (MRI) can then be used to assess myocardial edema and late gadolinium enhancement (LGE), applying the Lake Louise criteria when appropriate. If the diagnosis remains uncertain or the presentation is severe, endomyocardial biopsy (EMB) should be considered. Mechanistically, checkpoint blockade unleashes autoreactive, MYHCA-specific cytotoxic CD8^+^ T cells (perforin/granzymes) and an IFNγ→JAK/STAT program that expands CXCL9/CXCL10^+^ macrophages, amplifying injury—setting up a rationale for steroid-first care and escalation to pathway-targeted rescue. Given the potential for rapid clinical deterioration and life-threatening arrhythmias, early high-dose corticosteroids remain essential, with escalation reserved for severe or steroid-refractory cases. For steroid-refractory or rapidly progressive cases, escalation may include mechanism-aligned options such as CTLA4-Ig (abatacept) and/or JAK inhibition, while supportive immunomodulation, including intravenous immunoglobulin (IVIG) or plasma exchange (PLEX), may be considered in severe overlap presentations.

### 4.2 Gastrointestinal system—colitis under CTLA-4 blockade (±PD-1)

Immune-mediated colitis is classically associated with ipilimumab and is more frequent/severe with CTLA-4 + PD-1 combinations; PD-1 monotherapy can also cause diarrhea, abdominal pain, urgency, and bleeding (Dougan et al., 2021). Work-up must exclude infection (stool tests), use inflammatory indices (C-reactive protein (CRP), fecal calprotectin), and confirm with colonoscopy plus biopsies showing crypt abscesses, epithelial apoptosis, and mixed inflammatory infiltrates (Dougan et al., 2021; Haanen et al., 2022). Among these, stool frequency, fecal calprotectin, endoscopic severity, and early response to corticosteroids represent practical decision points for distinguishing mild disease from cases that may require earlier biologic escalation. In practice, fecal calprotectin, endoscopic severity, and response to initial corticosteroids can help identify patients who require earlier biologic escalation rather than prolonged empiric steroid exposure. Because gastrointestinal irAEs are among the most prevalent and practice-defining toxicities in routine ICI care, early symptom grading, stool-frequency assessment, and prompt distinction between uncomplicated diarrhea and true immune-mediated colitis are especially important in clinical decision-making (Abu-Sbeih et al., 2018). Accordingly, gastrointestinal toxicity remains a major real-world clinical priority because it is common, can escalate rapidly, and often directly affects treatment continuity.

### 4.3 Pulmonary system—pneumonitis under PD-1/PD-L1 inhibitors

Pneumonitis is one of the most clinically significant irAEs and, after myocarditis, a leading cause of ICI-related mortality. PD-1/PD-L1 agents (such as pembrolizumab, nivolumab, and atezolizumab) are linked to immune pneumonitis presenting with dry cough, exertional dyspnea, and low-grade fever, with high-resolution computed tomography (HRCT) patterns ranging from organizing pneumonia to diffuse alveolar damage or interstitial changes (Naidoo et al., 2017). Diagnostic priorities include prompt HRCT, exclusion of infectious etiologies and tumor progression via bronchoscopy/bronchoalveolar lavage (BAL) as indicated, and severity grading to guide inpatient care (Haanen et al., 2022). Biologically, tissue-resident T-cell activation and IFNγ-driven alveolar–interstitial inflammation engage myeloid amplifiers, supporting a grade-adapted corticosteroid strategy, with escalation to selected second-line immunomodulation (such as mycophenolate mofetil or IL-6R blockade in selected cases) when symptoms or imaging fail to improve (Franken et al., 2022; Brahmer et al., 2021; Lin et al., 2024).

### 4.4 Hepatobiliary system—hepatitis across PD-1/PD-L1 and CTLA-4

ICI hepatitis often presents as asymptomatic transaminase elevation but can progress to jaundice or coagulopathy; vigilance is crucial in HCC regimens (such as atezolizumab + bevacizumab) and with ipilimumab or PD-1 monotherapy (Dougan et al., 2021). Evaluation must exclude viral hepatitis, drug-induced liver injury, and tumor progression; autoimmune serologies can support immune mediation, and liver biopsy (interface hepatitis with lymphoplasmacytic infiltrates) refines diagnosis and stage (Zen and Yeh, 2019). Loss of hepatic immune tolerance via CD8^+^ T-cell and Kupffer-cell activation and IFNγ-chemokine circuits guides steroid induction and, when needed, antimetabolites or targeted cytokine-pathway blockade.

### 4.5 Neuromuscular system—Myositis (often with myocarditis/myasthenia gravis (MG) overlap)

ICI myositis—enriched under dual ICI—manifests as proximal weakness, myalgias, and CK elevation and warrants urgent co-assessment for myocarditis and myasthenia gravis due to “myo-cardio-neuro” overlap and respiratory involvement (Mehdi, 2019). Diagnostics include CK/troponin co-monitoring, electromyography (EMG), muscle biopsy, acetylcholine receptor (AChR)/muscle-specific kinase (MuSK) antibodies, and cardiac MRI/EMB if cardiac injury is suspected (Schneider et al., 2021). Shared skeletal/cardiac antigens likely drive cross-reactive T-cell cytotoxicity and IFNγ-programmed macrophage activation, supporting steroid pulses plus early escalation to mechanism-matched rescue for severe presentations.

### 4.6 Renal and others common irAEs—nephritis, dermatologic, and hematologic involvement

Renal irAEs (AIN > ATN) occur predominantly with PD-1/PD-L1 agents and present with rising creatinine and sterile pyuria or proteinuria; kidney biopsy can confirm interstitial nephritis and help guide therapy (Qu et al., 2021). Endocrine irAEs are among the most frequent toxicities encountered in routine practice and commonly include thyroid dysfunction, hypophysitis, adrenal insufficiency, and insulin-deficient diabetes mellitus (Wright et al., 2021). Their presentation may be subtle, with fatigue, headache, weight change, hypotension, or electrolyte abnormalities; accordingly, thyroid function tests, morning cortisol with or without adrenocorticotropic hormone (ACTH), glucose, and targeted pituitary evaluation should be integrated into routine assessment when clinically indicated. Once recognized, management usually depends more on timely hormone replacement and treatment interruption decisions than on prolonged high-dose immunosuppression. Dermatologic irAEs are among the earliest and most prevalent toxicities during ICI treatment and range from maculopapular rash and pruritus to lichenoid eruptions, vitiligo-like change, and bullous or pemphigoid-like disease requiring early dermatologic evaluation and biopsy (Sibaud, 2018). Mild cases may be managed with topical corticosteroids and antihistamines, whereas extensive, blistering, or steroid-refractory presentations warrant biopsy-guided escalation and specialist co-management. Together, endocrine and dermatologic toxicities account for a substantial proportion of the day-to-day irAE burden and therefore warrant proportionate emphasis in clinically oriented reviews. Hematologic toxicities (immune hemolysis, severe thrombocytopenia) are uncommon but emergent (Delanoy et al., 2019). Across these sites, organ-specific autoantigens, drug–antigen adducts, and convergent cytokine programs (such as JAK-STAT, TNF) shape risk, triage, and escalation beyond steroids when severe or refractory. Key diagnostic triggers and initial management/escalation options across organ systems are summarized in Table 1. The table distinguishes standard initial management from selected steroid-refractory escalation options, some of which remain organ-specific or emerging rather than universally established.

## 5 Clinical management of ICI-associated toxicities

### 5.1 Current treatment strategies

The cornerstone of ICI-associated toxicity management remains corticosteroid therapy, primarily methylprednisolone or prednisone. For mild to moderate irAEs, corticosteroids effectively reduce inflammation and suppress autoreactive immune responses. The standard initial dosage typically ranges from 0.5–2 mg/kg/day prednisone-equivalent depending on severity or the corresponding intravenous methylprednisolone dose, followed by a gradual taper over several weeks to avoid rebound toxicity (Haanen et al., 2022). Although corticosteroids remain the first-line intervention, their use is limited by potential side effects, such as hyperglycemia, osteoporosis, immunosuppression, and increased susceptibility to infections, as well as the possible impact on antitumor immune responses (Arbour et al., 2018). In this review, corticosteroids are regarded as the standard initial therapy for most clinically significant irAEs, whereas additional agents are discussed either as accepted second-line options in steroid-refractory settings or as emerging mechanism-guided approaches when evidence remains limited. This stratification is intended to support clearer clinical interpretation by distinguishing routine care from selected escalation strategies and emerging approaches.

In steroid-refractory cases, and in selected severe toxicities that fail to improve adequately with corticosteroids, additional immunosuppressive therapies may be required. Mycophenolate mofetil is frequently utilized as an accepted second-line option for steroid-resistant hepatitis and selected cases of colitis because of its selective inhibition of T-cell proliferation and favorable tolerability profile (Tay et al., 2017). Severe cardiac or neuromuscular irAEs, such as myocarditis or myositis, may in rare, highly refractory cases prompt consideration of more aggressive immunosuppressive therapies such as anti-thymocyte globulin (ATG) or alemtuzumab, an anti-CD52 antibody that rapidly depletes T cells; however, these approaches remain highly selected and investigational in practice, with evidence largely limited to case reports and small case series (Alouani et al., 2023). While effective, these agents carry significant risks, including profound immunosuppression, opportunistic infections, and secondary malignancies, emphasizing the need for careful patient selection and close monitoring (Esfahani et al., 2019).

### 5.2 Targeted therapy based on mechanistic insights

Immune toxicities are often driven by shared, re-activatable immunopathologic circuits that can manifest across different organs; therefore, management benefits from aligning organ-directed care with the dominant underlying program rather than treating each event as a fully independent, isolated process. A large share of severe myocarditis, colitis, and pneumonitis reflects an IFNγ–JAK/STAT–CXCL9/CXCL10 circuit in which cytotoxic T-cell activity amplifies myeloid CXCL9/CXCL10 and recruits CXCR3^+^ T cells; here, corticosteroids remain foundational, and selective JAK pathway blockade is a rational escalation when inflammation persists (mapping to Sections 4.1–4.3) (Daetwyler et al., 2024). Phenotypes dominated by clonal cytotoxic CD8^+^ T cells against shared/organ antigens—such as MYHCA in myocarditis or the myositis–myocarditis–MG triad (Sections 4.1/4.6)—may be candidates for mechanism-guided rescue with co-stimulation control (CTLA-4-Ig), and in fulminant settings may pair with JAK inhibition to interrupt both lymphocyte “spark” and myeloid “fuel” (Moslehi and Salem, 2022). For mucosal colitis enriched under CTLA-4 or combination therapy (Section 4.2), rapid control often follows TNF blockade, while gut-selective anti-integrin can restrain α4β7–MAdCAM-1 homing when hepatic injury or systemic TNF concerns coexist (Bergqvist et al., 2017). A subset of steroid-refractory pneumonitis/multisystem hyperinflammation appears IL-6-skewed; in selected refractory cases, IL-6R blockade (e.g., tocilizumab) may be considered in individual patients to attenuate the inflammatory cascade, although this approach is not currently standard of care.

Humoral-leaning patterns—neuromuscular overlap, immune cytopenias (Section 4.6)—call for IVIG or plasma exchange, with B-cell depletion to consolidate remission (Guidon et al., 2021). Endocrine irAEs typically represent durable gland loss (Section 4.4), so hormone replacement is central, reserving immunosuppression for brief inflammatory flares (de Filette et al., 2019). Immune hepatitis reflects breakdown of hepatic tolerance (Section 4.5); when steroids fail, antimetabolites (± calcineurin inhibition) are preferred and TNF blockade is generally avoided (Peeraphatdit et al., 2020). Re-challenge should be program-level: only after clinical resolution and biomarker normalization, with lower-risk regimens (such as avoiding dual blockade) and early-cycle monitoring for the prior pathway’s re-emergence (ECG/troponin after myocarditis; fecal calprotectin after colitis). Life-threatening myocarditis, severe neuromuscular overlap, and grade-4 pneumonitis/hepatitis typically preclude re-exposure (Simonaggio et al., 2019). This framework keeps decisions biologically grounded while not prescribing overly granular protocols, directly translating the organ phenotypes of Section 4 into targeted, mechanism-aware care.

### 5.3 Combinatorial ICI regimens and irAE risk

Combinatorial ICI regimens—including dual-checkpoint blockade and ICI-based combinations with other systemic therapies—have expanded rapidly across tumor types and treatment lines. While these strategies may enhance antitumor efficacy, regimen selection also materially shapes the incidence, timing, and organ spectrum of severe immune-related toxicities, and may complicate attribution when overlapping inflammatory adverse events occur. Notably, fulminant myocarditis has been reported early after ipilimumab–nivolumab, highlighting that rare but life-threatening events can be enriched in combination settings and warrant front-loaded surveillance and rapid multidisciplinary escalation when suspected (Johnson et al., 2016). In large randomized trials, dual ICI regimens (such as nivolumab plus ipilimumab) have demonstrated substantial rates of grade ≥3 treatment-related adverse events, underscoring the need for proactive monitoring, clear escalation thresholds, and regimen-specific risk communication (Motzer et al., 2018). Even in the adjuvant setting, regimen choice alters toxicity trade-offs: in resected stage III/IV melanoma, adjuvant nivolumab produced a markedly lower rate of grade 3–4 treatment-related adverse events than ipilimumab, underscoring that checkpoint class and dosing strategy are key determinants of severe immune toxicity (Weber et al., 2017; Weber et al., 2015). In addition, fatal toxic effects from ICIs—although uncommon overall—show regimen-dependent patterns, with higher fatality rates reported for PD-1/PD-L1 plus CTLA-4 combinations than for monotherapy, supporting systematic risk stratification and early diagnostic triage in combination regimens (Wang et al., 2018).

### 5.4 Emerging combination immunomodulation and personalized approaches

Combining mechanism-matched immunomodulators with corticosteroids is increasingly used to accelerate control of severe or steroid-refractory irAEs while minimizing prolonged exposure to broad immunosuppression. For example, abatacept or JAK inhibitors may be considered when T-cell–driven inflammation dominates, whereas IL-6R antagonists or anti-TNF agents provide biologic options for autoimmune-like phenotypes and steroid-sparing control (Jensen et al., 2025). Importantly, evidence also indicates that fatal irAEs—although rare overall—can be regimen-dependent and may occur early, particularly with PD-1/PD-L1 plus CTLA-4 combinations, highlighting the need for prompt escalation pathways once severe toxicity is suspected (Wang et al., 2018). The next step is patient-tailored management guided by immune profiling and clinical phenotype, integrating HLA risk markers and biomarker-guided escalation or de-escalation to align mechanisms with organ phenotypes and select the lowest-toxicity, highest-benefit intervention (Chennamadhavuni et al., 2022). By integrating molecular signals with clinical features, clinicians can shorten time-to-control, preserve antitumor efficacy, and move beyond steroid-centric care toward a precision, mechanism-informed irAE management paradigm (Refai, 2025).

## 6 Conclusion and future directions

ICIs have dramatically improved clinical outcomes in oncology, offering unprecedented therapeutic options for various malignancies. However, their clinical efficacy is frequently accompanied by irAEs, posing considerable challenges in oncology practice. Therefore, the safe and sustained use of ICIs critically depends on effective strategies to reduce irAEs without compromising antitumor efficacy. Recent research advances have elucidated critical molecular mechanisms underlying these toxicities, highlighting the breakdown of central and peripheral immune tolerance, autoreactive T cell proliferation, immune cell crosstalk, and cytokine-driven inflammatory pathways. These insights have significantly enhanced our understanding of ICI-associated adverse events, facilitating the development of targeted therapeutic interventions such as abatacept, JAK inhibitors, and other immunomodulatory agents, providing mechanism-guided alternatives to conventional corticosteroid-based treatments, with the potential to reduce cumulative immunosuppression and improve treatment continuity.

Despite these promising advances, clinical management of irAEs continues to face considerable challenges, notably in early diagnosis, risk stratification, and individualizing therapy. Moving forward, it is imperative to refine diagnostic methodologies through the validation of sensitive biomarkers, advanced imaging techniques, and integrated immune-profiling approaches. For example, immune-profiling approaches such as single-cell sequencing may allow timely intervention to prevent the onset or progression of refractory irAEs. In parallel, the identification and validation of predictive genetic markers, including specific HLA haplotypes and genetic polymorphisms associated with susceptibility to irAEs, may support proactive risk stratification and toxicity prevention strategies.

Looking ahead, precision medicine approaches focused on toxicity mitigation represent a critical frontier for optimizing the clinical benefit of ICIs while preserving patient safety. A multidisciplinary approach, combining insights from oncology, immunology, genetics, and clinical pharmacology, is indispensable to fully harness the potential of ICIs in cancer treatment while safeguarding patient safety and enhancing overall quality of life. Future research must prioritize these collaborative strategies to address existing gaps in knowledge, ultimately facilitating the development of safer, more effective immunotherapeutic interventions tailored to individual patient needs. Ultimately, shifting from reactive immunosuppression toward precision, mechanism-guided toxicity management will be essential for reducing adverse effects and enabling the long-term success of cancer immunotherapy.
